# Low Birth Weight due to Intrauterine Growth Restriction and/or Preterm Birth: Effects on Nephron Number and Long-Term Renal Health

**DOI:** 10.1155/2012/136942

**Published:** 2012-08-27

**Authors:** Vladislava Zohdi, Megan R. Sutherland, Kyungjoon Lim, Lina Gubhaju, Monika A. Zimanyi, M. Jane Black

**Affiliations:** ^1^Department of Anatomy and Developmental Biology, Faculty of Medicine, Nursing and Health Sciences, Monash University, Clayton, VIC 3800, Australia; ^2^Neuropharmacology, Baker IDI Heart and Diabetes Institute, 75 Commercial Road, Melbourne, VIC 3004, Australia; ^3^Preventative Health, Baker IDI Heart and Diabetes Institute, 75 Commercial Road, Melbourne, VIC 3004, Australia; ^4^Department of Anatomy and Pathology, School of Medicine, James Cook University, Townsville, QLD 4810, Australia

## Abstract

Epidemiological studies have clearly demonstrated a strong association between low birth weight and long-term renal disease. A potential mediator of this long-term risk is a reduction in nephron endowment in the low birth weight infant at the beginning of life. Importantly, nephrons are only formed early in life; during normal gestation, nephrogenesis is complete by about 32–36 weeks, with no new nephrons formed after this time during the lifetime of the individual. Hence, given that a loss of a critical number of nephrons is the hallmark of renal disease, an increased severity and acceleration of renal disease is likely when the number of nephrons is already reduced prior to disease onset. Low birth weight can result from intrauterine growth restriction (IUGR) or preterm birth; a high proportion of babies born prematurely also exhibit IUGR. In this paper, we describe how IUGR and preterm birth adversely impact on nephrogenesis and how a subsequent reduced nephron endowment at the beginning of life may lead to long-term risk of renal disease, but not necessarily hypertension.

## 1. Introduction

Low birth weight infants are at increased risk of renal disease. A reduced complement of nephrons at the beginning of life in infants that are intrauterine growth restricted (IUGR) and/or born preterm may be the cause of their long-term risk of renal disease. A reduced nephron endowment in low birth weight infants may also lead to vulnerability to hypertension in adulthood; however, experimental evidence suggests that they are not causally linked. In this paper we discuss how IUGR and preterm birth adversely impact on nephron endowment and how this has the potential to lead to deleterious effects on renal health.

The novelty of this paper is the emphasis on both preterm birth and IUGR leading to low birth weight; the effects of preterm birth on the kidney have only recently been described, and many of these studies have been undertaken in our laboratory. In this regard, much of the information reported in this paper has been derived from studies over the last decade in our laboratory.

## 2. Low Birth Weight due to IUGR and/or Preterm Birth

Low birth weight is defined as a birth weight of an infant less than 2500 grams at full term [1]. It can result from intrauterine growth restriction and/or preterm birth. IUGR is often a comorbidity of preterm birth and it is linked with induction of assisted and nonassisted premature delivery [2, 3].

### 2.1. Intrauterine Growth Restriction (IUGR)

**Figure 1:**
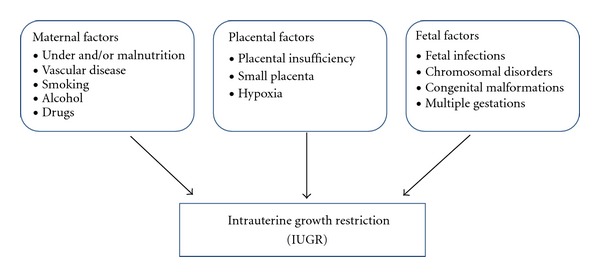
Intrauterine growth restriction (IUGR) can result from a variety of maternal, placental, and fetal factors, either individually or in combination.

The diagnosis of IUGR is assigned to infants with a birth weight and/or birth length below the 10th percentile for gestational age [4]. IUGR is multifactorial in origin and occurs in 4–10% of all term pregnancies [5]; as shown in Figure 1, IUGR is a manifestation of many possible fetal, placental, and maternal disorders [1]. Restriction of blood flow to the fetus is a common element to most IUGR pregnancies, resulting in lack of oxygen and nutrient delivery to the fetus [6, 7]. In developing countries, the major cause of IUGR is maternal undernutrition and/or malnutrition, whereas in developed countries, the majority of IUGR pregnancies are the result of placental insufficiency. The placenta is an important interface between mother and fetus, supplying oxygen and nutrients to the fetus and removing waste products. Hence, the maternal and fetal components of the placenta must be adequately perfused [8]. Placental insufficiency is mainly due to an inadequate vascular adaptation at the uteroplacental interface [9], which ultimately leads to fetal hypoxia and hypoglycaemia and subsequent fetal growth restriction [9, 10].

### 2.2. Preterm Birth

Preterm birth is defined as birth prior to 37 completed weeks of gestation [11]; it can be further subdivided according to severity [12]: near term (34–37 weeks gestation), moderately preterm (32-33 weeks gestation), very preterm (28–31 weeks gestation), and extremely preterm (<28 weeks gestation). Preterm birth currently accounts for 9.6% of births worldwide [13], with 12.3% of births in the USA [14] and 8.2% of births in Australia [15] being preterm. In developing countries, the incidence of preterm birth is generally higher; for example, the rates of preterm birth in southern Africa are 17.5% [13].

The proportion of preterm births is different for each gestational age category, with the majority of preterm births being near term; approximately 60% of preterm births are at 34–36 weeks gestation, 20% at 32-33 weeks, 15% at 28–31 weeks, and just 5% of preterm births are at less than 28 weeks gestation [12]. The incidence of preterm birth continues to rise worldwide [16–18]; this is largely attributed to the escalating use of assisted reproductive therapies over recent years (which has increased the number of multiple births) and to the rise in the number of deliveries following clinical intervention [12, 19]. More accurate assessments of gestational age at birth in recent times are also likely to have contributed to the number of neonates classified as being preterm [18].

The majority of preterm births (approximately 40%) occur due to spontaneous preterm labour, 25% occur following premature prelabour rupture of membranes, and 35% are clinically indicated [12]. The cause of preterm birth is often multifactorial in origin, with a large number of risk factors identified. These include maternal ethnicity, previous preterm birth, short time interval between pregnancies, low maternal body mass index, maternal smoking, multiple pregnancy, maternal stress, and also maternal medical conditions such as depression, cervical incompetence, thyroid disease, asthma, diabetes, and hypertension. The most significant contributor to preterm birth is intrauterine infection (including chorioamnionitis), which may be involved in up to 40% of preterm births [20, 21].

**Figure 2:**
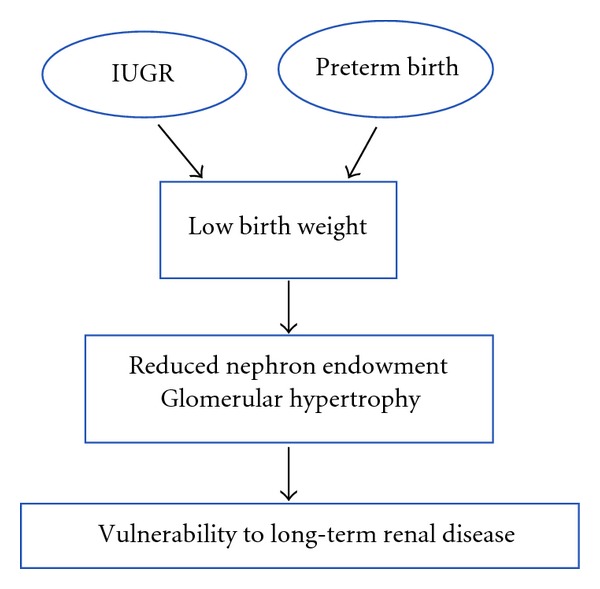
Flow diagram showing the consequences of intrauterine growth restriction (IUGR) and preterm birth which result in low birth weight. Low birth weight may have an adverse effect on nephron endowment and glomerular hypertrophy; together, these may increase the vulnerability of an individual born IUGR or preterm to long-term renal disease.

Importantly, both IUGR and preterm birth have the potential to adversely impact on kidney development; in the case of IUGR, this occurs *in utero* and in preterm infants, this occurs in the early neonatal period (Figure 2). This is particularly important in relation to the timing of nephrogenesis (the formation of nephrons) in the kidney.

## 3. Renal Development

The formation of the metanephric kidney commences at approximately week 5 of gestation with an outgrowth of cells from the caudal end of the nephric duct termed the ureteric bud [22, 23]. Reciprocal interactions subsequently occur between the ureteric bud and the metanephric mesenchyme, a mass of embryonic cells located at the base of the nephric cord. These interactions result in the differentiation of the mesenchyme to form nephrons and the growth and bifurcation of the ureteric bud to form the collecting ducts [22, 24].

The architecture of nephron arrangement within the human kidney is derived through the process of branching morphogenesis. As described by Osathanondh and Potter [23], 6–8 generations of ureteric branches form distal to the minor calyces by the completion of ureteric bud branching at approximately week 14-15 of gestation, with nephrons induced to differentiate at the ureteric bud tips (ampullae) from approximately the 9th week of gestation. After the cessation of active branching, 2–8 additional nephrons are induced to form at branch tips that have previously generated a nephron; in this way, generations of nephrons are formed in an arcade arrangement. The final nephrons formed after approximately 22 weeks gestation are attached individually to the terminal end of the ureteric branches; 8–10 generations of nephrons are formed in the human kidney by the completion of nephrogenesis [23, 25].

In the process of nephrogenesis, the induced mesenchyme condenses at the tip of the ureteric bud to form aggregates which then mature into renal vesicles. These subsequently progress into “comma shaped” and “S-shaped” bodies. The S-shaped body then fuses with a collecting duct and differentiates into a nephron. The renal corpuscle (comprised of the glomerulus and the Bowman's capsule) arises from the lower limb of the S-shaped body through a process of glomerulogenesis. Firstly, the inner cells of the lower cleft of the S-shaped body differentiate into the glomerular (visceral) podocytes, while the outer cells form the parietal podocytes which line the Bowman's capsule [22, 23, 26]. During the differentiation of podocytes, endothelial precursor cells from the mesenchyme invade the lower cleft and differentiate to form the capillary loop [27]. Both the podocyte epithelium and the developing endothelial cells actively synthesise the components required to form the shared glomerular basement membrane. The podocytes fuse with the developing glomerular basement membrane and further differentiate to form foot processes; the endothelium flattens and becomes fenestrated [27].

## 4. IUGR and Preterm Birth Adversely Impact on Nephrogenesis

Importantly, nephrogenesis is normally completed by 32–36 weeks gestation, with no new nephrons formed for the lifetime of the individual after this time [23, 28]. The nephrons are the functional units of the kidney, hence, the number of nephrons formed within the kidney at completion of nephrogenesis directly influences lifetime renal functional capacity and reserve [29]; it is essential, therefore, that adequate nephrogenesis is achieved at the very beginning of life. Autopsy studies over the past two decades have demonstrated that there is a wide range in nephron number in the human kidney [30–38]; overall, nephron number has been shown to range from approximately 200,000 to over 2 million per kidney [39]. The wide range in nephron number between individuals is likely attributed to differences in nephron endowment by the completion of nephrogenesis (which may be due to genetic and/or environmental factors), as well as differences in the exposure to secondary insults throughout life, which lead to loss of nephrons. In this regard, exposure to IUGR and/or preterm birth can negatively impact on nephrogenesis and thus adversely impact on nephron endowment at the beginning of life.

## 5. IUGR Leads to a Reduction in Nephron Endowment

A number of autopsy studies have reported a significant reduction in nephron number as a result of IUGR [31, 40, 41]. For example, it was found that nephron number in stillborn infants with IUGR was significantly reduced compared to infants that were appropriately grown-for-gestational age [40]. In another study, a linear relationship was reported between the number of glomeruli (and therefore nephrons) and birth weight in full-term neonates; neonates below the 10th percentile of birth weight had 30% fewer glomeruli than the neonates with birth weights above the 10th percentile [41]. In addition, many experimental studies in various animal models demonstrate that IUGR leads to a reduced nephron endowment at birth. Indeed, a low nephron endowment has been reported following naturally occurring IUGR due to twinning [42–44] and in experimentally induced IUGR offspring as a result of maternal nutritional deprivation [45, 46], maternal protein restriction [47–51], uterine artery ligation [52], and placental embolisation [10, 53]. In general, at the time of birth, kidney size is proportional to body size and nephron number is directly proportional to kidney size [54–56]; however, in the event that the growth restriction occurs late in gestation when nephrogenesis is complete (or nearing completion), this relationship does not exist [53].

## 6. Nephrogenesis Continues after Preterm Birth, but There Is Evidence of Glomerular Abnormalities and Acceleration of Renal Maturation

It has been proposed in previous studies that preterm birth leads to a reduced nephron endowment; Rodríguez et al. [57] found that preterm neonates (in particular those with a history of acute renal failure) had a significantly reduced number of glomerular generations compared to term-born controls. Additionally, a recently published experimental study demonstrated that a 20% reduction in nephron number occurred following premature delivery at 1-2 days prior to term birth in a preterm mouse model [58].

Preterm birth occurs at a time when nephrogenesis is often ongoing in the kidney of the infant. In our laboratory, we have clearly demonstrated in both baboon and human kidneys that nephrogenesis continues after preterm birth [54, 59]; observation of kidney sections from these preterm neonates clearly demonstrates an active nephrogenic zone with evidence of developing immature glomeruli. Furthermore, using unbiased stereological techniques, we have shown that the number of glomerular generations and the total number of nephrons increases in the postnatal period after preterm birth, thus, indicating that nephrogenesis continues in the extrauterine environment [54, 59]. Total nephron number appears to be within the normal range, albeit at the lower end of the normal range [54].

**Figure 3:**
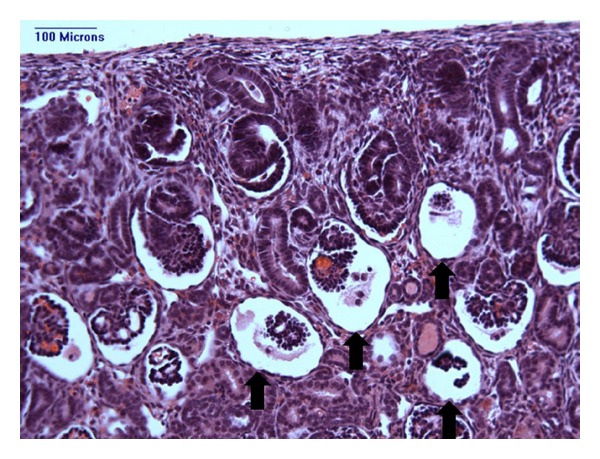
A representative photomicrograph of a histological renal section showing morphologically abnormal glomeruli (with enlarged Bowman's spaces and shrunken glomerular tufts) in the outer renal cortex of a preterm baboon kidney (indicated by arrows).

Of particular concern, many of the glomeruli in the outer cortex appeared markedly abnormal in some of the preterm kidneys; the number of abnormal glomeruli varied widely, from less than 1% to as high as 13% of glomeruli in the preterm human kidney [59] and 22% in the preterm baboon kidney [54, 55, 60]. As shown in Figure 3, the abnormal glomeruli exhibit a cystic morphology with a grossly enlarged Bowman's space. They are located within the outer renal cortex and are observed to be in an immature stage of development; the glomerular tuft is composed of an undifferentiated anlage of cells surrounded by a layer of podocytes with scant, if any, capillarisation [54, 55].

Our findings thus strongly suggest that it is the glomeruli formed postnatally in the extrauterine environment that are vulnerable to abnormalities. The abnormal glomerular morphology is characteristic of atubular glomeruli [61, 62], and if this is the case these glomeruli will never be functional. Hence, in infants with a high proportion of abnormal glomeruli, this will markedly impact on the number of functional nephrons at the beginning of life. Why some kidneys exhibit gross glomerular abnormalities whereas others are relatively unaffected is unknown, but likely relates to differences in the early postnatal care of the preterm infants whilst in the neonatal intensive care unit. For example, extremely preterm infants are known to experience extrauterine growth restriction (EUGR) since they generally do not achieve the normal rate of growth *ex utero* as that *in utero* [63, 64]. This is likely to have significant implications for ongoing nephrogenesis and consequently on renal function in adult life, therefore, in future studies, it is important to ascertain ways in which to achieve optimal renal development in the neonatal intensive care unit setting.

## 7. Glomerular Hypertrophy and Accelerated Renal Maturation in Preterm Neonates

Interestingly, we have shown a marked increase in the size of the preterm kidneys in the neonatal period which is not proportional to body size [54, 59]. In this regard, although absolute nephron number among preterm baboons was found to be within the normal range, on average the number of nephrons per gram of kidney tissue was significantly reduced in preterm baboon kidneys; 83,840 nephrons/gram were compared to 193,400 nephrons/gram in age-matched gestational controls [54]. These findings thus imply that there is glomerular hypertrophy (now clearly linked with renal pathology) and increased tubular mass within the renal cortex of the preterm kidneys. Quantitative examination of kidney tissue from preterm human neonates that was collected at autopsy subsequently confirmed that there was both glomerular hypertrophy and accelerated maturation of renal development in preterm kidneys after birth [59]. For example, there was a significant reduction in the width of the nephrogenic zone when compared to the kidneys from appropriately grown postconceptional age-matched stillborn infants [59]. Hence, although nephrogenesis was ongoing in the preterm kidneys, the reduced width of the nephrogenic zone implies that there was a diminished capacity to form new nephrons, probably due to acceleration in renal maturation. In support of this idea, we also showed a significant reduction in the proportion of the most immature glomeruli (vesicles) within the nephrogenic zone, indicative of fewer new glomeruli forming in the extrauterine environment.

In addition to accelerated maturation, there was also evidence of glomerular hypertrophy, probably due to the increased functional demands on the kidney after birth and/or an increase in renal blood flow. If this hypertrophy persists into later life, it is likely to lead to deleterious effects on renal function given that glomerular hypertrophy is linked to renal pathology in adulthood [39, 65]. Indeed, long-term hyperfiltration of hypertrophied glomeruli is proposed to lead to the development of glomerulosclerosis and subsequent glomerular loss [66, 67], thus, further reducing the functional capacity of the kidney.

## 8. Renal Blood Flow Increases at Birth

The glomerular hypertrophy and accelerated renal maturation in preterm infants are not surprising, given the marked change in hemodynamics at the time of birth and the increased functional demands on the kidney *ex utero*. Overall, the haemodynamic adaptations of the fetal kidney to extrauterine life involve transformation from a high vascular resistance fetal organ with low blood flow (primary blood flow to the inner cortex), into a high blood flow, low vascular resistance organ with primary blood supply to the outer renal cortex [68]. Blood flow to the kidneys is very low during fetal life, with a fetus at 10–20 weeks gestation only receiving 5% of cardiac output [69–71]. After birth, renal blood flow almost doubles, increasing to approximately 9% of cardiac output [69].

Importantly, studies in animal models with ongoing postnatal nephrogenesis have shown that after birth there initially remains a low blood flow to the outer renal cortex which is likely a protective mechanism to protect developing, immature glomeruli in this region of the kidney [72]. It is unknown, however, whether this protective pattern of blood distribution is also present in the kidney of the preterm human neonate, which is especially important given that these kidneys are structurally very immature and nephrogenesis is still ongoing in the outer renal cortex after birth.

## 9. Low Birth Weight Is Linked to Long-Term Vulnerability to Hypertension and Renal Disease

There are a number of studies both in humans and experimental models showing a link between low birth weight and long-term increases in blood pressure and susceptibility to the development of renal disease.

A systematic review of eighty studies, that had investigated the relationship between levels of blood pressure and birth weight, showed that in the majority of studies (conducted in either children, adolescents, or adults) blood pressure fell with increasing birth weight; there was an approximately 2 mmHg decrease in blood pressure for each increase in 1 kg of birth weight [73]. Although these effects on blood pressure appear relatively modest, they certainly have the potential to magnify cardiovascular risk, especially when other adverse life-style factors come into play. Indeed, only small changes in blood pressure can elevate cardiovascular risk; a relative rise of 2 mmHg in blood pressure is associated with 6% increased risk of coronary artery disease and a 15% increased risk of stroke [74].

In addition to long-term effects on blood pressure, there is substantial epidemiological data linking low birth weight with chronic kidney disease. In this regard, a recent meta-analysis of 31 relevant studies, conducted by White et al. [75], concluded that subjects born of low birth weight have a 70% greater risk of developing kidney disease. However, the authors did emphasise the need for additional well-designed population-based studies where confounders such as maternal health and socioeconomic factors are taken into account.

Most of the studies used in the meta-analyses described above have not separated low birth weight due to IUGR and preterm birth. Importantly, in this regard, there is now mounting epidemiological evidence linking preterm birth with the induction of hypertension in adults [76–84] and even in children [85–87]; there is an inverse relationship between gestational age at birth and level of blood pressure. For example, a recent study reported a 0.53 mmHg reduction in systolic blood pressure for every 1 week increase in gestational age at birth [83]. In addition, there are an emerging number of reports of adverse effects on renal function later in life in subjects that were born preterm (Table 1). However, it is to be noted that renal impairments are not a universal finding among studies involving preterm neonates which may be related to the more severe outcomes found among extremely preterm neonates born with a very low birth weight (VLBW; defined as birth weight less than 1000 grams). In support of this idea, Keijzer-Veen et al. [88] reported lower glomerular filtration rate and higher urinary albumin excretion in adults born extremely preterm who were also small-for-gestational age, compared to those born preterm and appropriate-for gestational age. Among children, Kwinta et al. [89] reported impaired renal function and reduced kidney volume among VLBW preterm neonates at 6-7 years of age and Zaffanello et al. [90] showed increased protein excretion among children born *extremely* LBW compared to children born VLBW. As discussed earlier, postnatal nutrition and weight gain may adversely impact on ongoing nephrogenesis and long-term renal function; in relation to this, Bacchetta et al. [91] have demonstrated lower glomerular filtration rate (albeit in the normal range) among children born very preterm (<30 weeks gestation) that were either IUGR or EUGR, compared to children with appropriate prenatal and postnatal growth. Furthermore, Kwinta et al. [89] have demonstrated a 33% reduced odds of renal impairment among children born very low birth weight if they gained weight during their stay at the neonatal intensive care unit.

The mechanisms leading to the increased risk of developing elevated blood pressure and vulnerability to long-term renal disease in low birth weight infants are currently unknown; a reduced nephron endowment at the beginning of life are postulated to play a role.

## 10. A Reduced Nephron Endowment in Early Life Is Not Causally Linked to the Development of Hypertension, but Likely Leads to Vulnerability to Long-Term Renal Disease

Convincing evidence from renal cross-transplantation studies directly implicates the kidney in the induction of essential/primary hypertension. For example, both human and experimental studies have shown that when the kidney from a hypertensive donor is transplanted into a normotensive recipient, it leads to the induction of hypertension in the recipient; the opposite occurs when the kidney from a normotensive donor is transplanted into a hypertensive recipient [97–100]. Further supporting the role of the kidney in the aetiology of hypertension, when a kidney from a young spontaneously hypertensive rat (prior to the onset of high blood pressure) is transplanted into a normotensive breed of rat, the recipient develops hypertension in early adulthood as is normally seen in the spontaneously hypertensive donor [97].

In the late 1980s, Brenner et al. [101] put forward the hypothesis that the fundamental renal abnormality that leads to hypertension is a reduced filtration surface area; this could be brought about by a reduction in the number of nephrons in the kidney and/or a decrease in renal filtration surface area per nephron [101]. Since, Brenner and colleagues initially put forward this hypothesis, there have been a multitude of experimental studies investigating the effects of early life insults (such as IUGR) on nephron endowment at the beginning of life [48, 53, 56, 102] and the long-term effects on blood pressure [48–50, 52, 103–107]. Although there has been considerable experimental support for the concept that a reduced nephron endowment leads to hypertension, the findings are far from universal. For instance, in the rat model of maternal protein restriction Langley-Evans et al. [48], Sahajpal and Ashton [104], Woods et al. [108, 109] have observed long-term elevations in blood pressure in the presence of a reduced nephron endowment, whereas studies from our and other laboratories show no rise in blood pressure in adulthood [49–51, 110]. A number of studies suggest that the discrepancies in findings between studies relate to differences in methodologies associated with the measurement of blood pressure; it appears that the IUGR offspring has an exaggerated stress response, so when the measurement of blood pressure leads to stress in the rats, they have an elevation in blood pressure [105, 111]. Indeed, in many of the early studies, blood pressure was measured only at one time point in unconditioned rats using tail-cuff plethysmography; these papers are repeatedly cited in the literature as evidence of IUGR leading to an elevation in blood pressure, but it is now becoming well-accepted that an elevated stress response was probably the mediator. In fact, recent studies from the same laboratory show a decrease in blood pressure when it is measured by telemetry, but an elevation of blood pressure is apparent when blood pressure is measured by tail-cuff plethysmography [105]. A commonly quoted human study is that of Keller et al. [35] where a significant reduction in the number of nephrons per kidney was found in an autopsy study of middle-aged white subjects from Germany who suffered from primary hypertension when compared to normotensive subjects. It is important to keep in mind when interpreting the findings of such studies that hypertension can lead to renal injury [112, 113] and so the reduction in nephron endowment may have been a result of the hypertension, not the cause. However, this does not appear to be the case in the study by Keller et al. [35]; the proportion of sclerotic glomeruli in the hypertensive kidneys was low (<5-6%).

Importantly, in our laboratory, we have conducted a number of studies which have addressed the Brenner hypothesis [49, 53, 114, 115]. In our IUGR models, the data generated could neither refute nor support Brenner's hypothesis. Using gold standard stereological techniques, we counted the number of nephrons as well as measured renal filtration surface area within the IUGR kidney [49, 53]. In all instances of reduced nephron endowment, we observed a compensatory increase in glomerular size with a concomitant increase in glomerular filtration surface area per glomerulus, such that renal filtration surface area was not compromised [49, 53]. Hence, in our model of IUGR, since renal filtration surface area was not compromised, it was not surprising (based on Brenner's hypothesis) that blood pressure was not affected [49]. Using an alternative approach, we have undertaken cross-breeding studies between spontaneously hypertensive rats and the normotensive Wistar Kyoto strain [114]; in the F2 progeny (where there is a random segregation of the SHR and WKY genes), the offspring displayed a wide range of blood pressures. To examine whether there was a direct corollary between the level of blood pressure and nephron number and/or renal filtration surface area, we conducted regression analyses in the F2 progeny; in these studies, we found no significant correlations between level of blood pressure and nephron endowment or renal filtration surface area [114].

Hence, overall the experimental evidence now clearly indicates that a reduced nephron endowment at the beginning of life does not necessarily lead to hypertension. However, it is conceivable that when the functional reserve of nephrons is severely reduced, the glomeruli will eventually reach the limits of physiological compensatory hypertrophy and pathological mechanisms will come into play; elevations in blood pressure may then also ensue. Indeed, prolonged hyperfiltration of hypertrophied glomeruli can ultimately lead to glomerulosclerosis and eventual glomerular loss [116, 117]. Since it is loss of glomeruli throughout life which accelerates risk and ultimately leads to end stage renal disease, it is likely that vulnerability to disease will be increased when the functional reserve is reduced prior to disease onset. In this regard, it has been postulated that a congenital nephron deficit acts as an initial insult to the kidney, which when combined with subsequent postnatal insults (secondary hits), leads to an exacerbated decline in renal function [118]. There are a number of experimental studies that support this idea, whereas other studies do not. For example, IUGR with a concomitant reduction in nephron endowment has been shown to lead to more severe glomerulosclerosis in a model of mesangioproliferative glomerulonephritis in rats [119]. In addition, in our laboratory, we have shown that the kidneys of IUGR rat offspring, with a reduced nephron endowment, are more vulnerable to the infusion of advanced glycation end products (AGEs) [50]. This implies that IUGR kidneys with a congenital nephron deficit may be more vulnerable to the induction of diabetes, given that AGE formation is markedly elevated with hyperglycemia and their accumulation in tissues is linked to the pathogenesis of end-organ damage in diabetes [120]. Consistent with this idea, the progression of renal disease is faster in patients with a single kidney [121]. We have subsequently gone on to examine the effect of induction of streptozotocin diabetes in adulthood in our IUGR maternal protein restriction rat model [122]. Interestingly, we found that the marked impairment of hyperglycemia on renal function was not exacerbated in the IUGR rats compared to controls [122]. This is possibly due to compensatory glomerular hypertrophy in the IUGR offspring. When Jones et al. [123] examined the effect of induction of streptozotocin diabetes on renal structure in IUGR rats, they found that IUGR rats had a greater proportional increase in renal size compared to non-IUGR diabetic rats; following insulin treatmen,t the renal hypertrophy was reduced but the glomeruli remained hypertrophied in the IUGR rats [123]. A number of experimental studies have also examined the effect of feeding a high-salt diet to IUGR rats as a secondary renal insult to rats with a congenital nephron deficit; in general, it appears that the IUGR kidneys can adequately cope with an increased salt load [49, 51]. In this regard, findings from our laboratory showed no evidence of salt-sensitive hypertension in IUGR rats when fed a high-salt diet in adulthood; analysis of the kidneys showed compensatory glomerular hypertrophy in the IUGR rats with the congenital nephron deficit such that renal filtration surface area was not different when compared to controls. However, when nephron number is markedly reduced this is not likely to be the case; administration of a high-salt diet in uninephrectomised IUGR rats has been shown to lead to a marked reduction in glomerular filtration rate and elevation in blood pressure [124].

## 11. Summary

This paper has shown how IUGR and preterm birth can adversely impact on the number of nephrons in the kidney and lead to glomerular hypertrophy; this in turn can lead to the induction of pathological processes within the kidney when the glomeruli reach their limits of compensation. A reduced nephron endowment does not necessarily lead to hypertension but renders the kidney vulnerable to long-term renal disease. Since IUGR is often a comorbidity of preterm birth, it is likely that individuals who were born IUGR and preterm will be particularly vulnerable to secondary renal insults.

## Figures and Tables

**Table 1:** Summary of the studies that have examined blood pressure and renal function in children and adults that were born preterm.

**Blood pressure**				
Authors	Number and sex of participants	Weeks gestational age at birth	Age of subjects at examination (years)	Major findings in participants born preterm
Cooper et al. [83]	7847 Males and females	<37	44–45	Higher DBP Inverse association between GA and systolic BP

Bonamy et al. [86]	60 Males and females	≤30	7–12	Higher SBP and heart rate Lower dermal capillary density

Dalziel et al. [92]	458 Males and females	32–35	30	Higher SBP Insulin resistance

Keijzer-Veen et al. [79]	82 Males and females	<32	20	Higher SBP

Lawlor et al. [82]	386,485 Males	35–44	18	Both birthweight and GA inversely correlated with systolic blood pressure

Bonamy et al. [87]	66 Females	23–34	16.5	Higher brachial and aortic blood pressures (DBP and SBP) Narrower abdominal aortas Increased vascular resistance

Hack et al. [81]	195 Males and females	30	20	Higher systolic blood pressure

Johansson et al. [80]	404,306 Males	24–43	18	Higher SBP

Keijzer-Veen et al. [78]	596 Males and females	<32	19	Increased prevalence of high blood pressure

Doyle et al. [77]	270 Males and females	24–36	18.6	Higher SBP and DBP

Stevenson et al. [85]	128 Males and females	26–37	15	Higher SBP

Kistner et al. [76]	50 Females	28–32	23–26	Higher SBP and DBP

Siewert-Delle and Ljungman [84]	430 Males	30–37	49	SBP inversely related to gestational age

**Renal function**				
Authors	Number and sex of participants	Weeks of gestational age at birth (range)	Age of subjects at examination (years)	Major findings in participants born preterm

Kwinta et al. [89]	116 Males and females	26–29	6–7	Increased serum cystatin-C levels Decreased kidney volume No difference in microalbuminuria

Zaffanello et al. [90]	69 Males and females	26–31	5–6	Urine *α*1-microglobulin levels significantly increased in ELBW versus VLBW No difference in GFR or microalbuminuria in ELBW versus VLBW

Bacchetta et al. [91]	50 Males and females	<37	7.6	Lower GFR in IUGR and EUGR No difference in microalbuminuria

Rakow et al. [93]	105 Males and females	<32	9–12	No difference in GFR or microalbuminuria Smaller kidney volume

Keijzer-Veen et al. [79]	82 Males and females	<32	20	Greater filtration fraction in preterm AGA Increased microalbuminuria in preterm SGA

Iacobelli et al. [94]	48 Males and females	<37	6.3–8.2	Increased microalbuminuria No difference in GFR

Keijzer-Veen et al. [88]	422 Males and females	<32	19	Lower GFR in SGA Increased microalbuminuria in SGA

Rodríguez-Soriano et al. [95]	40 Males and females	23–35	6.1–12.4	Lower GFR No difference in kidney size or blood pressure

Kistner et al. [76]	50 Females	28–32	23–26	No difference in GFR or effective renal plasma flow No difference in microalbuminuria

Vanpee et al. [96]	34 Males and females	25–30	8	No difference in GFR or effective renal plasma flow No difference in microalbuminuria

GA: gestational age; DBP: diastolic blood pressure; SBP: systolic blood pressure; GFR: glomerular filtration rate; AGA: appropriate weight for gestational age; SGA: small for gestational age; ELBW: extremely low birth weight (<1kg); VLBW: very low birth weight (1–1.5 kg); IUGR: intrauterine growth restriction; EUGR: extrauterine growth restriction.
